# Influence of the Mechanical Properties of Elastoplastic Materials on the Nanoindentation Loading Response

**DOI:** 10.3390/ma13214842

**Published:** 2020-10-29

**Authors:** Huanping Yang, Wei Zhuang, Wenbin Yan, Yaomian Wang

**Affiliations:** School of Metallurgical Engineering, Xi’an University of Architecture and Technology, Xi’an 710055, China; zhuangwei1112@163.com (W.Z.); yanwenbin0609@163.com (W.Y.)

**Keywords:** nanoindentation, mechanical property, finite element method, elastoplastic

## Abstract

The nanoindentation loading response of elastoplastic materials was simulated by the finite element method (FEM). The influence of the Young’s modulus *E*, yield stress *σ_y_*, strain hardening exponent *n* and Poisson’s ratio *ν* on the loading response was investigated. Based on an equivalent model, an equation with physical meaning was proposed to quantitatively describe the influence. The calculations agree well with the FEM simulations and experimental results in literature. Comparisons with the predictions using equations in the literature also show the reliability of the proposed equation. The investigations show that the loading curvature *C* increases with increasing *E*, *σ_y_*, *n* and *ν*. The increase rates of *C* with *E*, *σ_y_*, *n* and *ν* are different for their different influences on the flow stress after yielding. It is also found that the influence of one of the four mechanical parameters on *C* can be affected by the other mechanical parameters.

## 1. Introduction

**Figure 1:**
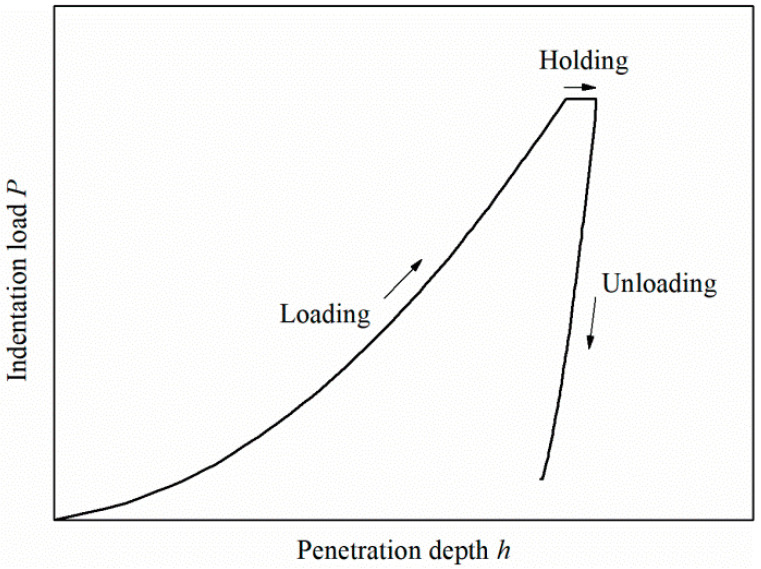
Typical *P*-*h* curve.

Nanoindentation has become a powerful quantitative method for characterizing the mechanical properties of materials on a small scale. It is widely used in composite materials, multiphase alloy, nanostructured materials, thin films, and coatings [1,2,3,4,5,6,7,8]. During the course of the indentation, a record of the load and the corresponding depth of penetration can be made. A typical indentation load to penetration depth (*P*–*h*) curve of elastoplastic materials using a Berkovich indenter is shown in Figure 1. It consists of loading, holding and unloading segments. Mechanical properties such as the hardness, elastic modulus, strain hardening exponent, and yield stress can be extracted from the curve through different methods [9,10,11,12,13,14,15,16,17].

The loading *P–h* curves of the elastoplastic materials with a sharp indenter can be described by Kick’s law [18,19,20,21] in Equation (1):

(1) $$P={\mathrm{Ch}}^{2}$$

where *C* is the loading curvature. For a given load *P*, the elastoplastic materials with high flow stress during indentation show a relatively small penetration depth *h* and therefore exhibit a high value of *C*. The value of *C* is determined by the mechanical properties of the indented material. Understanding the influence of the mechanical properties on *C* has great importance for evaluating nanoindentation measurements.

The following Equation (2) has been given to express the influence of the yield stress $\sigma_{y}$, Young’s modulus E and Poisson’s ratio ν [22,23,24]:

(2) $$C=M_{1}\sigma_{0.29}\left(1+\frac{\sigma_{y}}{\sigma_{0.29}}\right)\left[M_{2}+ln\left(\frac{E^{*}}{\sigma_{y}}\right)\right]$$

where $M_{1}$ and $M_{2}$ are constants, $E^{*}$ is the effective elastic modulus and can be expressed by $E^{*}={\left[\left(1-\nu^{2}\right)/E+\left(1-\nu_{i}^{2}\right)/E_{i}\right]}^{-1}$ in which the subscript *i* denotes indenter, and $\sigma_{0.29}$ is the stress corresponding to the characteristic plastic strain of 0.29 for the indented material in uniaxial compression.

By using dimensional analysis and FEM simulation, Dao et al. [25] proposed the following Equation (3):

(3) $$C=\sigma_{0.033}\left\{-1.131{\left[ln\left(\frac{E^{*}}{\sigma_{0.033}}\right)\right]}^{3}+13.635{\left[ln\left(\frac{E^{*}}{\sigma_{0.033}}\right)\right]}^{2}-30.594\ln\left(\frac{E^{*}}{\sigma_{0.033}}\right)+29.267\right\}$$

where $\sigma_{0.033}$ is the stress corresponding to a plastic strain of 0.033.

According to equivalent energy principle, Chen et al. [26] derived Equation (4) to express the influence of mechanical properties on loading curvature for conical indentation with a semi-vertical angle of 70.3°:

(4) $$C=110.829561\frac{\sigma_{y}^{1-n}E^{n}}{1+n}0.0536^{n}0.893665^{500\frac{\sigma_{y}}{E}}$$

Other equations describing the influence of the Young’s modulus, hardness and other mechanical properties on the loading curvature have also been proposed [27,28,29]. These formulas are helpful for investigating the nanoindentation loading response of the indented materials. To elucidate the influence mechanism of mechanical properties more clearly, the equation should have clear physical meaning. Because the loading curvature *C* is affected by the stress response of indented materials and the mechanical properties of the materials can be described by Young’s modulus *E*, yield stress $\sigma_{y}$, strain hardening exponent *n* and Poisson’s ratio *ν*, and the expression for *C* should explicitly include these four parameters.

In this study, the finite element method (FEM) was used to investigate the nanoindentation loading response of elastoplastic materials. The influences of the Young’s modulus *E*, yield stress *σ_y_*, strain hardening exponent *n* and Poisson’s ratio *ν* on the loading curvature *C* were analyzed. An equation to describe the influences was proposed. The quantitative relations of *C* to the mechanical properties were discussed in detail.

## 2. FE Model

**Figure 2:**
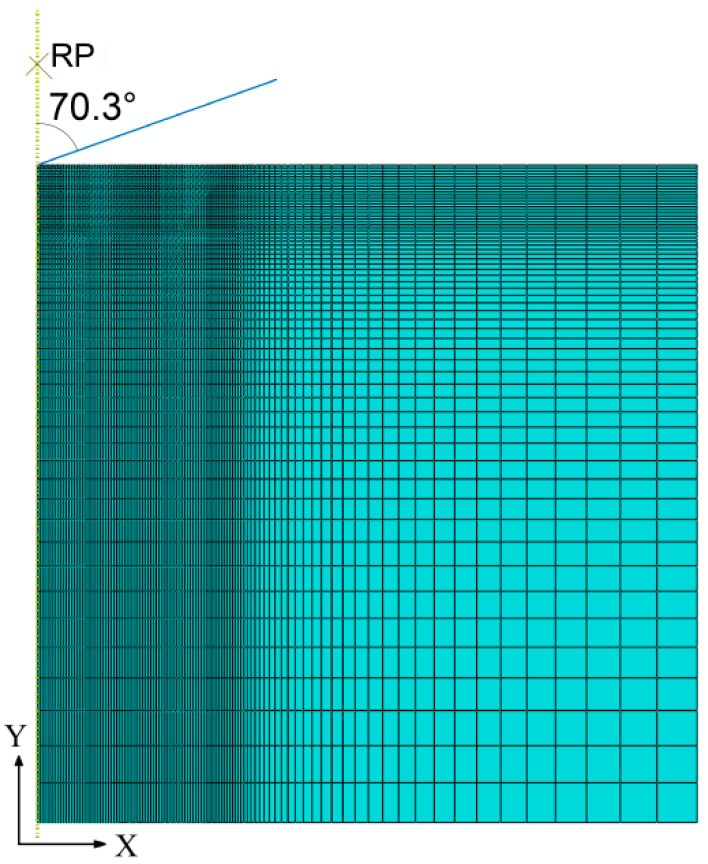
FE model of nanoindentation.

A conical indenter with an apex angle θ = 70.3° has the same projected area–depth functions as the standard Berkovich indenter used in the nanoindentation tests [21,25]. Studies have shown that the computational *P*–*h* responses of the conical and Berkovich indentations were virtually identical [25,30]. Considering the symmetry of the conical indenter, an axisymmetric two-dimensional FE model was developed to simulate the indentation procedure. The simulated *P*–*h* curves using the two-dimensional model showed good agreement with the experiment ones [7,21,25,31]. In this study, an axisymmetric deformable two-dimensional 10 μm × 10 μm planar FE model, shown in Figure 2, was established to simulate the elastoplastic response of materials during nanoindentation. The indented material was meshed by linear quadrilateral elements CAX4 with gradual refinement near the contact region. The indenter was modeled as a rigid body and the contact between the indenter and the material was frictionless in the simulation [7,21,25,31,32,33]. The indenter was constrained to a reference point (RP) rigidly. A concentrated force with the maximum value of 50 mN was applied to the RP in the general static analysis step.

For the boundary condition, the x displacements of the nodes along the Y axis were fixed. Both the x displacement and y displacement of those nodes on the bottom face were fixed. The RP can move only along the Y axis. The nanoindentations were simulated for *E*,$\;\sigma_{y}$ *n*, and ν ranges of 60–220 GPa, 500–1200 MPa, 0.15–0.5, and 0.25–0.35, respectively.

Once the calculation was completed, the records of the displacement and the corresponding load for the RP at different increment steps can be obtained. Thus, the simulated nanoindentation *P*–*h* curve during loading was obtained. The loading curvature *C* can be calculated through the least squares method.

## 3. Results

### 3.1. Influence of the Young’s Modulus

**Figure 3:**
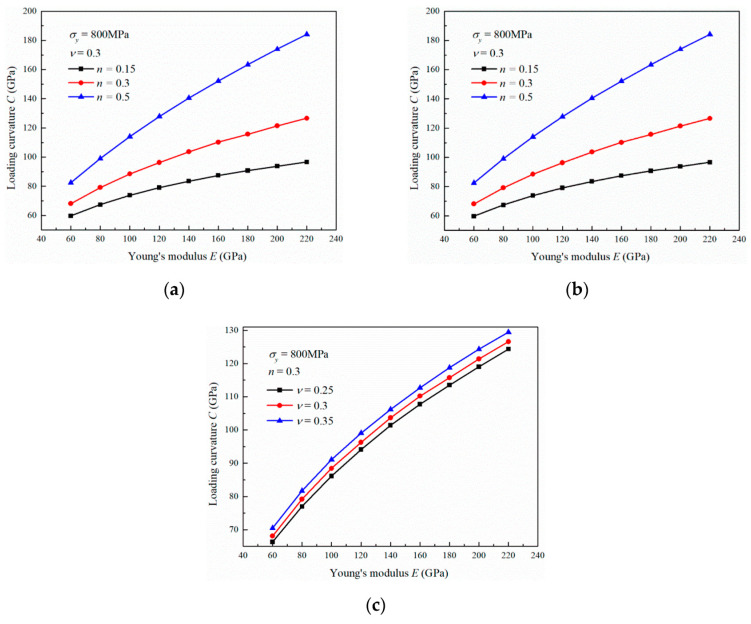
Influence of the Young’s modulus on the loading curvature with (**a**) different yield stresses, (**b**) different strain hardening exponents, and (**c**) different Poisson’s ratios.

With increasing Young’s modulus, the material shows a higher stress level for resisting elastoplastic deformation during indentation. Therefore, the penetration depth *h* becomes small, and the loading curvature *C* becomes large, as shown in Figure 3. The loading curvature *C* increases quickly with the Young’s modulus *E* in the range of 60 to 220 GPa, and the increase rate $\frac{\partial C}{\partial E}$ decreases gradually. From Figure 3a,b, it can be found that the loading curvature exhibits a faster increase with the Young’s modulus as the yield stress and strain hardening exponent increase. The Poisson’s ratio also shows a similar influence on the relation between the loading curvature and Young’s modulus, although it is not obvious, as shown in Figure 3c. The values of loading curvature are very close with the increase of Poisson’s ratio from 0.25 to 0.35 for the materials with Young’s modulus of 60 GPa, and the differences between the loading curvatures became slightly larger as the Young’s modulus increased to 220 GPa. 

### 3.2. Influence of the Yield Stress

**Figure 4:**
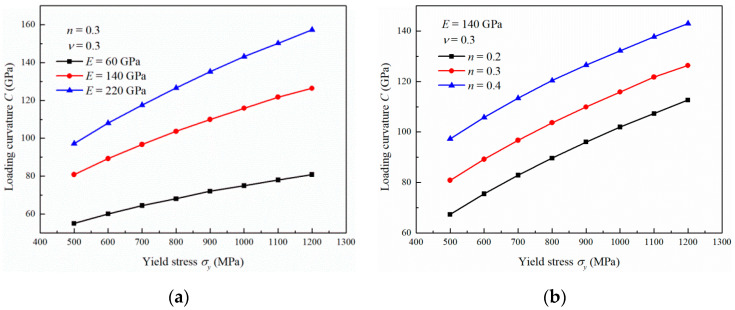

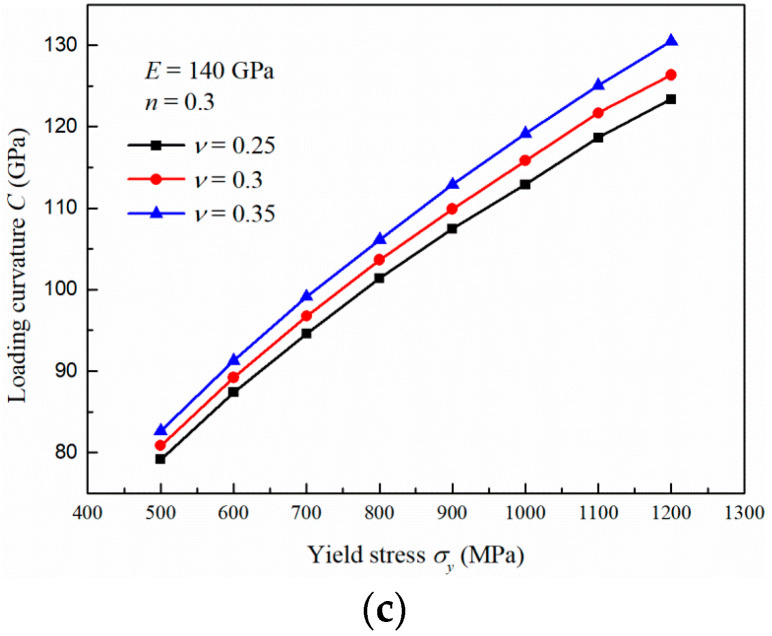
Influence of the yield stress on the loading curvature with (**a**) different Young’s moduli, (**b**) different strain hardening exponents and (**c**) different Poisson’s ratios.

The influence of the yield stress on the nanoindentation loading curvature is shown in Figure 4. The loading curvature increases substantially with the yield stress in the range of 500 to 1200 MPa. The increase rate $\frac{\partial C}{\partial \sigma_{y}}$ decreases gradually with yield stress. It can also be seen that the loading curvature shows a faster increase with the yield stress as the Young’s modulus and Poisson’s ratio increase. However, with increasing strain hardening exponent, the increase rate $\frac{\partial C}{\partial \sigma_{y}}$ seems to remain constant, as shown in Figure 4b.

### 3.3. Influence of the Strain Hardening Exponent

**Figure 5:**
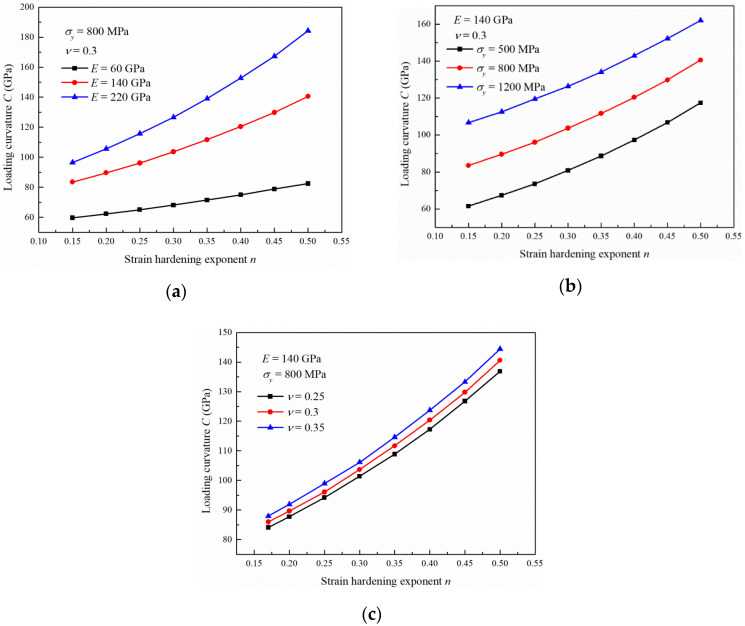
Influence of the strain hardening exponent on the loading curvature with (**a**) different Young’s moduli, (**b**) different yield stresses and (**c**) different Poisson’s ratios.

Similar to the Young’s modulus and yield stress, the strain hardening exponent shows an obvious influence on the loading curvature, as shown in Figure 5. A substantial increase in *C* with *n* can be noted. However, the increase rate $\frac{\partial C}{\partial n}$ increases gradually with increasing *n*, which is different from the variations in Figure 3 and Figure 4. This indicates that the hardening exponent increment can increase the flow stress more significantly compared with Young’s modulus and yield stress. As the Young’s modulus and Poisson’s ratio increase, *C* exhibits a faster increase with *n*. However, the variation in $\sigma_{y}$ shows no effects on the increase rate $\frac{\partial C}{\partial n}$, as shown in Figure 5b.

### 3.4. Influence of the Poisson’s Ratio

**Figure 6:**
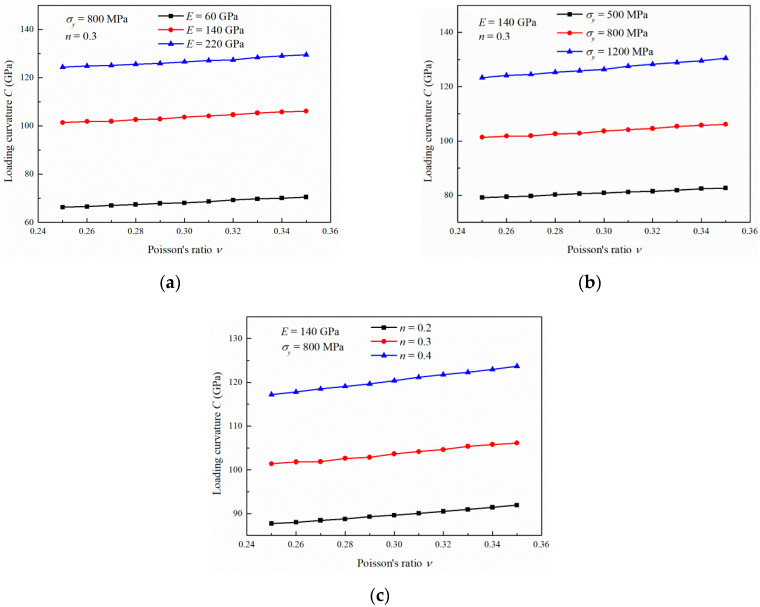
Influence of the strain hardening exponent on the loading curvature with (**a**) different Young’s moduli, (**b**) different yield stresses and (**c**) different Poisson’s ratios.

The relations between *ν* and *C* are shown in Figure 6. The values of *C* show a slight increase with increasing *ν* in the range of 0.25 to 0.35. This indicates that the values of *ν* of the materials have a weak influence on *C* compared with *E*, $\sigma_{y}$ and *n*. It can also be found that the increase rate $\frac{\partial C}{\partial \nu }$ is almost stable with increasing *ν*. The variations in $\frac{\partial C}{\partial \nu }$ with *ν* are not greatly influenced by the change in *E*, $\sigma_{y}$ and *n*.

## 4. Discussion

The value of *C* is affected by the stress level of the indented materials, which is determined by the indenter and the material mechanical properties. Additionally, it is shown that *C* has a stress unit from Equation (1). Therefore, we can suppose that *C* is proportional to an equivalent stress $\sigma_{E}$ of the materials during indentation.

The typical stress–strain relation beyond $\sigma_{y}$ for metal materials can be written as Equation (5) [14,25]:

(5) $$\sigma =\sigma_{y}{\left(1+\frac{E}{\sigma_{y}}\varepsilon_{p}\right)}^{n}=\sigma_{y}{\left(\frac{\varepsilon_{y}+\varepsilon_{p}}{\sigma_{y}/E}\right)}^{n}=\sigma_{y}{\left(\frac{\varepsilon }{\varepsilon_{y}}\right)}^{n}$$

where *ε_p_* is the nonlinear part of the total strain ε and $\varepsilon_{y}$ is the initial yield strain corresponding to $\sigma_{y}$. It can be found that the flow stress σ is an amplification of $\sigma_{y}$ by a coefficient ${\left(\frac{\varepsilon }{\varepsilon_{y}}\right)}^{n}$. 

**Figure 7:**
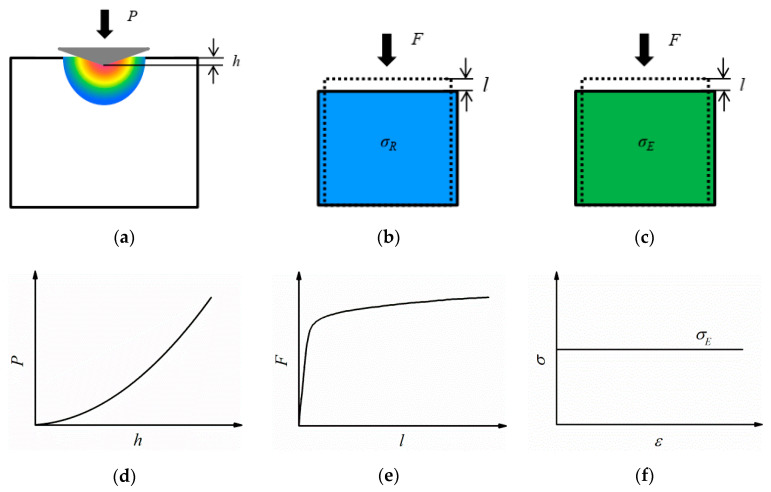
Illustrations of the equivalent model of nanoindentation: (**a**) nanoindentation, (**b**) RVE with varying stress during plastic deformation, (**c**) RVE with constant stress during plastic deformation, (**d**) typical loading *P*–*h* response, (**e**) typical *F*–*l* relation, and (**f**) mechanical response of the RVE in (**c**).

Considering a representative volume element (RVE) which is compressed by a load *F* (shown in Figure 7b), the typical relationship between the *F* and displacement *l* induced by compression for most metal materials is shown in Figure 7e. In addition, the variation of flow stress $\sigma_{R}$ with strain of the RVE can be expressed by Equation (5). To obtain the nanoindentation loading *P–h* relationship as shown in Figure 7d, the stress $\sigma_{R}$ must be amplified. According to Equation (5) and the FEM simulation results, Equation (6) is suggested to express the stress $\sigma_{R}$:

(6) $$\sigma_{R}=\left(1+\nu \right){\left(\frac{\varepsilon }{\varepsilon_{y}}\right)}^{n}\sigma =\left(1+\nu \right)\frac{\sigma^{2}}{\sigma_{y}}=\left(1+\nu \right)\sigma_{y}{\left(1+\frac{E}{\sigma_{y}}\varepsilon_{p}\right)}^{2n}$$

Because the loading curvature *C* is a measure of the stress level of the indented materials and it is a constant, the equivalent stress $\sigma_{E}$, which is equal to $\sigma_{R}$ at a certain strain ${\bar{\varepsilon }}_{p}$, is used to illustrate the stress level of the RVE shown in Figure 7c. This means that the stress of the RVE during compression is constant at different plastic strains as shown in Figure 7f.

Then, the influence of mechanical properties on loading curvature *C* can be expressed explicitly by Equation (7):

(7) $$C=A\sigma_{E}=A\left(1+\nu \right)\sigma_{y}{\left(1+\frac{E}{\sigma_{y}}{\bar{\varepsilon }}_{p}\right)}^{2n}$$

where *A* is a coefficient. According to the FEM simulations using different combinations of *E*, $\sigma_{y}$, *n* and *ν*, it is found that the ${\bar{\varepsilon }}_{p}$ value of 0.008 can give the smallest standard deviation of *A*. Therefore, ${\bar{\varepsilon }}_{p}=0.008$ and A=57.6566 in Equation (7).

From Equation (7), the increase rate of *C* with *E*, $\sigma_{y}$, *n*, and *ν* can be obtained and given by Equations (8)–(11). It is easy to see that $\frac{\partial C}{\partial E}>0$, $\frac{\partial C}{\partial n}>0$, and $\frac{\partial C}{\partial \nu }>0$. For most metal materials, n is smaller than 0.5, so $\frac{\partial C}{\partial \sigma_{y}}>0$. These results are in agreement with the FEM simulation results where *C* increases with *E*, $\sigma_{y}$, *n* and *ν*.

(8) $$\frac{\partial C}{\partial E}=2A\left(1+\nu \right)n{\bar{\varepsilon }}_{p}{\left(1+\frac{E}{\sigma_{y}}{\bar{\varepsilon }}_{p}\right)}^{2n-1}$$

(9) $$\frac{\partial C}{\partial \sigma_{y}}=A\left(1+\nu \right)\left(1+\frac{E}{\sigma_{y}}{\bar{\varepsilon }}_{p}-\frac{2nE}{\sigma_{y}}{\bar{\varepsilon }}_{p}\right){\left(1+\frac{E}{\sigma_{y}}{\bar{\varepsilon }}_{p}\right)}^{2n-1}$$

(10) $$\frac{\partial C}{\partial n}=2A\left(1+\nu \right)\sigma_{y}{\left(1+\frac{E}{\sigma_{y}}{\bar{\varepsilon }}_{p}\right)}^{2n}ln\left(1+\frac{E}{\sigma_{y}}{\bar{\varepsilon }}_{p}\right)$$

(11) $$\frac{\partial C}{\partial \nu }=A\sigma_{y}{\left(1+\frac{E}{\sigma_{y}}{\bar{\varepsilon }}_{p}\right)}^{2n}$$

Based on Equation (8), the second derivative is given as Equation (12):

(12) $$\frac{\partial^{2}C}{\partial E^{2}}=2A\left(1+\nu \right)n\left(2n-1\right)\frac{{\bar{\varepsilon }}_{p}^{2}}{\sigma_{y}}{\left(1+\frac{E}{\sigma_{y}}{\bar{\varepsilon }}_{p}\right)}^{2n-2}<0$$

This indicates that $\frac{\partial C}{\partial E}$ decreases with *E,* as shown in Figure 3. Similarly, we can obtain the following Equations (13)–(15):

(13) $$\frac{\partial^{2}C}{\partial \sigma_{y}^{2}}=2A\left(1+\nu \right)\frac{{\left(E{\bar{\varepsilon }}_{p}\right)}^{2}}{\sigma_{y}^{3}}n\left(2n-1\right){\left(1+\frac{E}{\sigma_{y}}{\bar{\varepsilon }}_{p}\right)}^{2n-2}<0$$

(14) $$\frac{\partial^{2}C}{\partial n^{2}}=4A\left(1+\nu \right)\sigma_{y}{\left(1+\frac{E}{\sigma_{y}}{\bar{\varepsilon }}_{p}\right)}^{2n}ln^{2}\left(1+\frac{E}{\sigma_{y}}{\bar{\varepsilon }}_{p}\right)>0$$

(15) $$\frac{\partial^{2}C}{\partial \nu^{2}}=0$$

This means that $\frac{\partial C}{\partial \sigma_{y}}$ decreases with $\sigma_{y}$, $\frac{\partial C}{\partial n}$ increases with n, and $\frac{\partial C}{\partial \nu }$ is constant with ν. These results agree well with the FEM simulation results.

The loading curvature *C* is affected by the stress response of the material during indentation. From Equation (6), it can be found that the increase in the flow stress after yielding gradually becomes slower because *n* is smaller than 0.5 for most metal materials. Although the flow stress can be increased to some extent with increasing *E* and $\sigma_{y}$, the increase rates of *C* with *E* and $\sigma_{y}$ still show a gradual decrease. As the strain hardening exponent *n* increases, both the stress and its rate of increase can be increased. Therefore, the increase rate of *C* with *n* shows an increasing increment. For the influence of the Poisson’s ratio ν, it can be found that the stress increases with increasing ν, but the increase rate is invariable. Therefore, the increase rate of *C* with ν is constant.

We can also obtain the second derivative as follows.

(16) $$\frac{\partial^{2}C}{\partial E\partial \sigma_{y}}=\frac{\partial^{2}C}{\partial \sigma_{y}\partial E}=2A\left(1+\nu \right)n\left(1-2n\right){\left(1+\frac{E}{\sigma_{y}}{\bar{\varepsilon }}_{p}\right)}^{2n-2}\frac{E{\bar{\varepsilon }}_{p}^{2}}{\sigma_{y}^{2}}>0$$

(17) $$\frac{\partial^{2}C}{\partial E\partial n}=\frac{\partial^{2}C}{\partial n\partial E}=2A\left(1+\nu \right){\bar{\varepsilon }}_{p}{\left(1+\frac{E}{\sigma_{y}}{\bar{\varepsilon }}_{p}\right)}^{2n-1}\left[1+2nln\left(1+\frac{E}{\sigma_{y}}{\bar{\varepsilon }}_{p}\right)\right]>0$$

(18) $$\frac{\partial^{2}C}{\partial E\partial \nu }=\frac{\partial^{2}C}{\partial \nu \partial E}=2An{\bar{\varepsilon }}_{p}{\left(1+\frac{E}{\sigma_{y}}{\bar{\varepsilon }}_{p}\right)}^{2n-1}>0$$

(19) $$\frac{\partial^{2}C}{\partial \sigma_{y}\partial \nu }=\frac{\partial^{2}C}{\partial \nu \partial \sigma_{y}}=A{\left(1+\frac{E}{\sigma_{y}}{\bar{\varepsilon }}_{p}\right)}^{2n-1}\left(1+\frac{E}{\sigma_{y}}{\bar{\varepsilon }}_{p}-\frac{2nE}{\sigma_{y}}{\bar{\varepsilon }}_{p}\right)>0$$

(20) $$\frac{\partial^{2}C}{\partial n\partial \nu }=\frac{\partial^{2}C}{\partial \nu \partial n}=2A\sigma_{y}{\left(1+\frac{E}{\sigma_{y}}{\bar{\varepsilon }}_{p}\right)}^{2n}ln\left(1+\frac{E}{\sigma_{y}}{\bar{\varepsilon }}_{p}\right)>0$$

From Equation (16), it can be known that $\frac{\partial C}{\partial E}$ increases with increasing $\sigma_{y}$. This agrees with the simulation results shown in Figure 3a, where $\frac{\partial C}{\partial E}$ increases as $\sigma_{y}$ increases from 500 to 1200 MPa. The equation also indicates that $\frac{\partial C}{\partial \sigma_{y}}$ increases with increasing *E*, which is consistent with the results shown in Figure 4a, where $\frac{\partial C}{\partial \sigma_{y}}$ increases with increasing *E* from 60 to 220 GPa. The variations in *C* described by Equations (17)–(20) also agree well with the simulation results.

**Figure 8:**
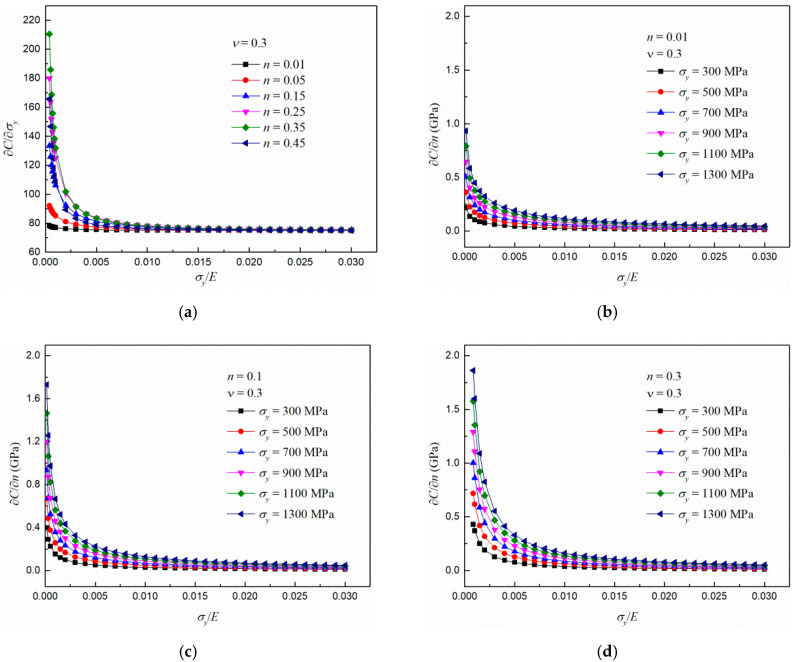
Influence of $\frac{\sigma_{y}}{E}$ on the increase rates (**a**) $\frac{\partial C}{\partial \sigma_{y}}$ with varing *n*, $\frac{\partial C}{\partial n}$ with varing yield stress as (**b**) *n* = 0.01, (**c**) *n* = 0.1 and (**d**) *n* = 0.3.

According to Equation (9), the variations of $\frac{\partial C}{\partial \sigma_{y}}$ with $\frac{\sigma_{y}}{E}$ as *n* increases from 0.01 to 0.45 are shown in Figure 8a. It can be found that the values of $\frac{\partial C}{\partial \sigma_{y}}$ reaches stable and are very close as $\frac{\sigma_{y}}{E}$ is bigger than 0.005. In addition, this is accordance with this simulation results shown in Figure 4b. According to Equation (10), the variations of $\frac{\partial C}{\partial n}$ with $\frac{\sigma_{y}}{E}$ as $\sigma_{y}$ increasing from 300 to 1300 MPa are shown in Figure 8b–d. It can be seen that $\frac{\partial C}{\partial n}$ decreases quickly with $\frac{\sigma_{y}}{E}$ and reaches a stable value for different combinations of $\sigma_{y}$ and *n*. In addition, the stable values of $\frac{\partial C}{\partial n}$ are close with varying $\sigma_{y}$. This agrees with the results shown in Figure 5b.

Based on the above analysis, Equation (7) can provide a reasonable description of the influence of the material mechanical properties on the nanoindentation loading curvature. These material parameters exhibit different effects on the loading response. *C* increases with *E*, $\sigma_{y}$, *n*, and *ν* because of the positive value of its first derivative. Owing to $\frac{\partial^{2}C}{\partial E^{2}}<0$ and $\frac{\partial^{2}C}{\partial \sigma_{y}^{2}}<0$, the increase rates $\frac{\partial C}{\partial E}$ and $\frac{\partial C}{\partial \sigma_{y}}$ decrease gradually with increasing *E* and $\sigma_{y}$, respectively. The increase rate $\frac{\partial C}{\partial n}$ increases gradually with *n* because $\frac{\partial^{2}C}{\partial n^{2}}>0$. The increase rate $\frac{\partial C}{\partial \nu }$ is constant with increasing ν because $\frac{\partial^{2}C}{\partial \nu^{2}}=0$.

The influence of *E* on *C* can be intensified with increasing $\sigma_{y}$, *n* and ν since $\frac{\partial^{2}C}{\partial \sigma_{y}\partial E}>0$, $\frac{\partial^{2}C}{\partial n\partial E}>0$ and $\frac{\partial^{2}C}{\partial \nu \partial E}>0$, respectively. This means that $\frac{\partial C}{\partial E}$ is increased with increasing $\sigma_{y}$, *n* and ν. Similarly, the influence of $\sigma_{y}$ on *C* can be intensified with increasing *E* and ν because $\frac{\partial^{2}C}{\partial E\partial \sigma_{y}}>0$ and $\frac{\partial^{2}C}{\partial \nu \partial \sigma_{y}}>0$. However, the increase rate $\frac{\partial C}{\partial \sigma_{y}}$ almost remains constant with variation of *n* as $\frac{\sigma_{y}}{E}$ is bigger than 0.005. The influence of *n* on *C* can be intensified with increasing *E* and ν because $\frac{\partial^{2}C}{\partial E\partial n}>0$ and $\frac{\partial^{2}C}{\partial \nu \partial n}>0$. However, the increase rate $\frac{\partial C}{\partial n}$ does not change with $\sigma_{y}$. The influence of ν on *C* intensifies with increasing *E*, $\sigma_{y}$, and *n* because $\frac{\partial^{2}C}{\partial E\partial \nu }>0$, $\frac{\partial^{2}C}{\partial \sigma_{y}\partial \nu }>0$ and $\frac{\partial^{2}C}{\partial n\partial \nu }>0$.

**Figure 9:**
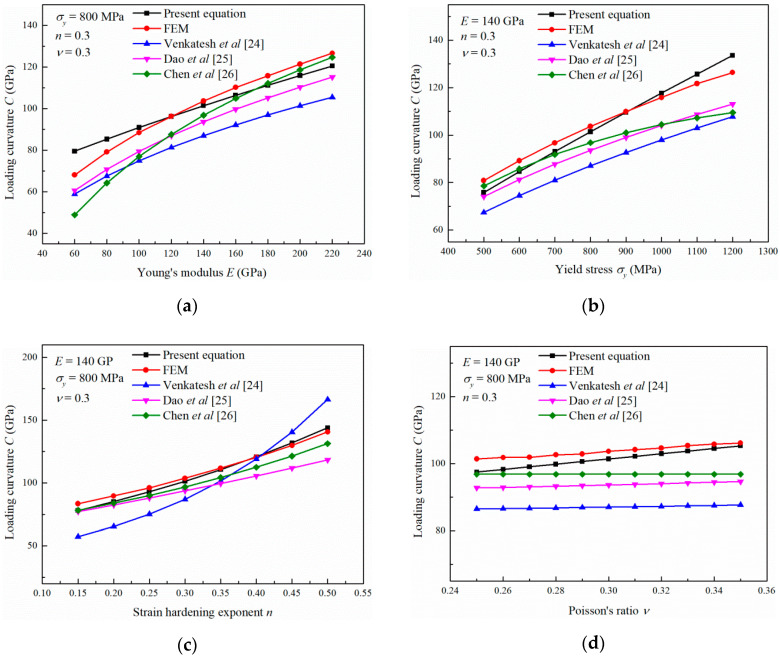
Comparisons of the variations in the loading curvatures with the (**a**) Young’s modulus, (**b**) yield stress, (**c**) strain hardening exponent and (**d**) Poisson’s ratio.

Compared with the equations in the literature, Equation (7) proposed in this study has a clear physical meaning. The proposed equation quantitatively and reasonably describes the influence of *E*, $\sigma_{y}$, *n*, and *ν* on the nanoindentation loading response of elastoplastic materials. The calculation results using Equation (7) and using Equations (2)–(4) given in the literature, as well as the FEM simulation results, are illustrated in Figure 9. The results calculated by the present equation show good agreement with the FEM simulations. In addition, the values of *C* obtained by the present equation are close to the ones calculated through the equations in the literature. Comparisons between the predicted results with the experimental values in literature are summarized in Table 1. It is noted that the proposed equation can give a good prediction. Therefore, Equation (7) can be used to conveniently and credibly elucidate the nanoindentation loading response of elastoplastic materials.

## 5. Conclusions

1. The equation proposed in the present study can quantitatively and reasonably describe the effects of the mechanical properties of elastoplastic materials on the nanoindentation loading curvature. The calculated results agreed well with the FEM simulations and experimental results in literature.
2. The nanoindentation loading curvature *C* increases with increasing Young’s modulus *E*, yield stress $\sigma_{y}$, strain hardening exponent *n* and Poisson’s ratio ν because the equivalent stress increases. The increase rates $\frac{\partial C}{\partial E}$ and $\frac{\partial C}{\partial \sigma_{y}}$ decrease gradually with increasing *E* and $\sigma_{y}$, respectively. The increase rate $\frac{\partial C}{\partial n}$ increases gradually with *n*, and the increase rate $\frac{\partial C}{\partial \nu }$ remains constant with increasing ν.
3. The influence of *E* on *C* can be intensified with increasing $\sigma_{y}$, *n* and ν. The influence of $\sigma_{y}$ on *C* can be intensified with increasing *E* and ν. *n* has little effect on the increase rate $\frac{\partial C}{\partial \sigma_{y}}$. The influence of *n* on *C* can be intensified with increasing *E* and ν. However, $\sigma_{y}$ does not affect the increase rate $\frac{\partial C}{\partial n}$. The influence of ν on *C* can be intensified with increasing *E*, $\sigma_{y}$, and *n.*

## Figures and Tables

**Table 1:** Comparisons of predictions with experiment results in literature.

Material	Mechanical Property				*C* (GPa)				
	*E* (GPa)	*σ_y_* (MPa)	*n*	*ν*	Experiment	Calculation			
						Present Equation	Venkatesh et al. [24]	Dao et al. [25]	Chen et al. [26]
Al 6061-T6511 [25]	66.8	284	0.08	0.33	27.4 ^a^	25.8	19.0	27.8	28.1
Al 7075-T651 [25]	70.1	500	0.122	0.33	42.7 ^a^	46.1	31.8	43.8	42.3
Al 6061-T6 [31]	70.6	331.7	0.081	0.33 ^b^	33.9 ^c^	30.0	21.7	31.6	31.8
AISI 1010 [31]	209.6	210.6	0.249	0.3 ^d^	46.9 ^c^	47.0	38.4	46.3	47.5
AISI 1045 [31]	210.3	337.1	0.202	0.3 ^d^	59.3 ^c^	52.1	42.8	55.6	57.7
Al 2024-T351 [32]	68	360	0.08	0.33	36.0 ^e^	32.0	22.7	33.1	33.0

^a^ Averaged from 6 tests; ^b^ Ref. [25]; ^c^ Averaged from 9 tests; ^d^ Ref. [18]; ^e^ Estimated by fitting the loading curve.
